# Rates of resistance and heteroresistance to newer β-lactam/β-lactamase inhibitors for carbapenem-resistant Enterobacterales

**DOI:** 10.1093/jacamr/dlae048

**Published:** 2024-03-21

**Authors:** Christina K Lin, Alex Page, Sarah Lohsen, Ali A Haider, Jesse Waggoner, Gillian Smith, Ahmed Babiker, Jesse T Jacob, Jessica Howard-Anderson, Sarah W Satola

**Affiliations:** Department of Medicine, Emory University School of Medicine, Atlanta, GA, USA; Division of Infectious Diseases, Department of Medicine, Emory University School of Medicine, Atlanta, GA, USA; Division of Infectious Diseases, Department of Medicine, Emory University School of Medicine, Atlanta, GA, USA; Division of Infectious Diseases, Department of Medicine, Emory University School of Medicine, Atlanta, GA, USA; Division of Infectious Diseases, Department of Medicine, Emory University School of Medicine, Atlanta, GA, USA; Division of Infectious Diseases, Department of Medicine, Emory University School of Medicine, Atlanta, GA, USA; Georgia Emerging Infections Program, Atlanta, GA, USA; Atlanta Veterans Affairs Medical Center, Decatur, GA, USA; Division of Infectious Diseases, Department of Medicine, Emory University School of Medicine, Atlanta, GA, USA; Department of Pathology and Laboratory Medicine, Emory University School of Medicine, Atlanta, GA, USA; Division of Infectious Diseases, Department of Medicine, Emory University School of Medicine, Atlanta, GA, USA; Georgia Emerging Infections Program, Atlanta, GA, USA; Division of Infectious Diseases, Department of Medicine, Emory University School of Medicine, Atlanta, GA, USA; Georgia Emerging Infections Program, Atlanta, GA, USA; Division of Infectious Diseases, Department of Medicine, Emory University School of Medicine, Atlanta, GA, USA; Georgia Emerging Infections Program, Atlanta, GA, USA; Department of Pathology and Laboratory Medicine, Emory University School of Medicine, Atlanta, GA, USA

## Abstract

**Background:**

Heteroresistance (HR), the presence of antibiotic-resistant subpopulations within a primary isogenic population, may be a potentially overlooked contributor to newer β-lactam/β-lactamase inhibitor (BL/BLI) treatment failure in carbapenem-resistant Enterobacterales (CRE) infections.

**Objectives:**

To determine rates of susceptibility and HR to BL/BLIs ceftazidime/avibactam, imipenem/relebactam and meropenem/vaborbactam in clinical CRE isolates.

**Methods:**

The first CRE isolate per patient per year from two >500 bed academic hospitals from 1 January 2016 to 31 December 2021, were included. Reference broth microdilution (BMD) was used to determine antibiotic susceptibility, and population analysis profiling (PAP) to determine HR. Carbapenemase production (CP) was determined using the Carba NP assay.

**Results:**

Among 327 CRE isolates, 46% were *Enterobacter cloacae*, 38% *Klebsiella pneumoniae* and 16% *Escherichia coli*. By BMD, 87% to 98% of CRE were susceptible to the three antibiotics tested. From 2016 to 2021, there were incremental decreases in the rates of susceptibility to each of the three BL/BLIs. HR was detected in each species–antibiotic combination, with the highest rates of HR (26%) found in *K. pneumoniae* isolates with imipenem/relebactam. HR or resistance to at least one BL/BLI by PAP was found in 24% of CRE isolates and 65% of these had detectable CP.

**Conclusion:**

Twenty-four percent of CRE isolates tested were either resistant or heteroresistant (HR) to newer BL/BLIs, with an overall decrease of ∼10% susceptibility over 6 years. While newer BL/BLIs remain active against most CRE, these findings support the need for ongoing antibiotic stewardship and a better understanding of the clinical implications of HR in CRE.

## Introduction

Infections with carbapenem-resistant Enterobacterales (CRE) have been associated with high rates of antibiotic failure and mortality,^1–3^ necessitating the development and use of newer β-lactam/β-lactamase inhibitor (BL/BLI) combinations such as ceftazidime/avibactam, imipenem/cilastatin-relebactam and meropenem/vaborbactam. Prior work analysing CRE in the USA has demonstrated rates of susceptibility greater than 80% to newer BL/BLI combinations and 46%–87% of CRE were found to have carbapenemase genes. However, whether susceptibility patterns have changed over time has not been well studied.^4–6^

In addition, heteroresistance (HR), or the presence of antibiotic-resistant subpopulations within a primary isogenic susceptible population, may be a potential contributor to treatment failure. However, HR is not routinely assessed in the clinical microbiology laboratory.^7^ The reported frequency of HR in CRE (HR-CRE) isolates to colistin, imipenem and meropenem has varied widely in prior studies, ranging from 0% to 100%.^8^ Several of these studies pre-selected isolates for HR testing, therefore making it difficult to estimate the overall rate of HR.^9,10^ The aims of this study were: (i) to create an antibiogram of CRE isolates’ susceptibility rates to new BL/BLIs from 2016 to 2021 and (ii) to determine rates of HR to imipenem/relebactam, ceftazidime/avibactam and meropenem-vaborbactam in a convenience sample of CRE clinical isolates collected from a large academic healthcare network. A secondary aim was to test for and compare the frequency of phenotypic detection of carbapenemase production (CP) between susceptible, heteroresistant (HR) and resistant CRE isolates.

## Materials and methods

### Study population and isolate collection

From 1 January 2016 to 31 December 2021, CRE isolates from two >500 bed academic hospitals were collected as part of the CDC-funded Georgia Emerging Infections Program’s (GA EIP) Multi-site Gram-Negative Surveillance Initiative (MuGSI). GA EIP conducts active population and laboratory-based surveillance for CRE, defined as resistant to doripenem, imipenem, meropenem (MIC > 2 mg/L) or ertapenem (MIC > 1 mg/L) and isolated as part of routine clinical care in participating hospitals in metropolitan Atlanta, GA, USA. Cases are identified by routine queries of laboratory automated testing instruments.^11^ Only carbapenem-resistant *Enterobacter cloacae*, *Klebsiella pneumoniae* and *Escherichia coli*, as identified by the hospital microbiology laboratory via MALDI-TOF MS and VITEK 2 GN74 (bioMérieux, Durham, NC, USA) were included in this study. Only the first isolate per patient per year was included for those with multiple cultures recovering the same species.

### Susceptibility testing with broth microdilution (BMD)

MICs of ceftazidime/avibactam (susceptible ≤ 8/4 mg/L, resistant ≥ 16/4 mg/L), imipenem/relebactam (susceptible ≤ 1/4 mg/L, intermediate = 2/4 mg/L, resistant  ≥ 4/4 mg/L) and meropenem/vaborbactam (susceptible  ≤ 4/8 mg/L, intermediate = 8/8 mg/L, resistant ≥ 16/8 mg/L), were determined using reference BMD per CLSI guidelines.^12,13^

### HR population analysis profiling (PAP)

PAP testing for HR to ceftazidime/avibactam, imipenem/relebactam and meropenem/vaborbactam was conducted per a modified protocol^14,15^ of the microdilution plating method as follows: bacterial strains were streaked from frozen glycerol stock samples onto Mueller–Hinton II plates and grown overnight at 37°C. Per CLSI guidelines for antibiotic susceptibility testing, isolates were subcultured from the freezer stock twice prior to testing by PAP for HR. Single colonies were selected to inoculate a 48-well plate containing 300 µL of CAMHB, and cultures were grown for approximately 18 h at 37°C in a shaking incubator at 500 rpm. Cultures were serially diluted 1:10 in CAMHB over a series of seven dilutions. Aliquots of 7.5 µL of the dilutions were spotted on Mueller–Hinton II agar plates with no antibiotic, as well as 2-fold increasing concentrations of antibiotics. The following concentrations of antibiotic were used for ceftazidime/avibactam: 0.025/4, 0.5/4, 1/4, 2/4, 4/4, 8/4, 16/4 and 32/4 mg/L; for imipenem/relebactam: 0.25/4, 0.5/4, 1/4, 2/4, 4/4 and 8/4 mg/L; and for meropenem/vaborbactam: 0.025/8, 0.5/8, 1/8, 2/8, 4/8, 8/8, 16/8 and 32/8 mg/L. Plates were incubated for 20–24 h at 37°C before colonies were counted. For HR determination, log-transformed ratios of cfu/mL of colonies surviving on drug-containing agar relative to cfu/mL without the presence of drug were calculated at the breakpoints (16/4 mg/L for ceftazidime/avibactam, 4/4 mg/L for imipenem/relebactam and 16/8 mg/L for meropenem/vaborbactam). Strains were considered HR if they displayed a log-transformed ratio of cfu/mL growing on the antibiotic breakpoint plate divided by the cfu/mL in the starting inoculum with no antibiotic <−0.3 (less than 50% survival) but above the limit of detection (usually more than 0.0001% survival). PAPs were performed in duplicate and for each antibiotic. Isolates that were identified as HR were validated a second time. An isolate with a resistant MIC by BMD can be defined as HR by PAP.

### CP and Amber class identification

CP and Ambler class identification was determined using a Carba NP method outlined by Dortet *et al*.^16^ with the following modifications: instead of two 10 μL inoculation loops of bacteria being mixed with 200 μL of two BPER II (Tris HCl lysis buffer), two 1 μL inoculation loops of bacteria were mixed into 525 μL of BPER II; indicator solutions and lysate volumes were reduced to 20 μL each and mixed in a 384-microwell plate; indicator solution/lysate suspensions were not incubated at 37°C, but monitored at room temperature with optical measurements at 560 nm wavelength being measured every 5 min over 30 min on a SpectraMax iD3 Microplate Reader and then reevaluated at 2 h for visible colour changes. These modifications have previously been evaluated against the standard protocol and produced the same results for carbapenemase detection and Ambler class distinction.

### Statistical analysis

We determined the percent susceptibility and resistance for each BL/BLI by dividing the number of susceptible or resistant CRE isolates (as determined by BMD) by the total number of CRE isolates tested per species per year. We calculated the proportion of HR in a similar method except that the number of HR isolates was obtained from PAP results. All calculations were done in Microsoft Excel.

### Ethics

The GA EIP surveillance, data collection and analysis were approved by the Emory University Institutional Review Board (IRB#00089004).

## Results

Among the 327 CRE isolates included, 151 (46%) were *E. cloacae*, 123 (38%) *K. pneumoniae* and 53 (16%) *E. coli*. Almost half (154/327; 47%) of the cultures were from urine, 87/327 (27%) were from sterile sites including blood, 44/327 (13%) were from the respiratory tract, and 42/327 (13%) were from other non-sterile sites (Table 1 and Table S1, available as Supplementary data at *JAC-AMR* Online).

**Table 1.** Culture site of CRE isolates by species in two academic hospitals in Atlanta, GA, 2016–21

	Culture site, *n* (%)			
CRE species	Urine	Sterile^a^	Respiratory	Other^b^
*E. coli* (*n* = 53; 16%)	31 (58)	13 (25)	3 (6)	6 (11)
*K. pneumoniae* (*n* = 123; 38%)	60 (49)	35 (28)	22 (18)	6 (5)
*E. cloacae* (*n* = 151; 46%)	63 (42)	39 (26)	19 (13)	30 (20)
Total (*n* = 327)	154 (47)	87 (27)	44 (13)	42 (13)

^a^Sterile included blood, bone, deep tissue/internal abscess, pleural fluid, peritoneal fluid and other normally sterile sites.

^b^Other included other, non-sterile culture sites such as wounds, drainage and non-sterile tissue.

Most CRE isolates were susceptible to all three BL/BLIs (ceftazidime/avibactam, imipenem/relebactam and meropenem/vaborbactam) by standard antibiotic susceptibility testing (AST), with 7/9 (78%) of the species–antibiotic combinations having a >90% susceptibility rate (Table S3). Carbapenem-resistant *E. cloacae* had the highest rates of susceptibility to all three BL/BLIs; 148/151 (98%) were susceptible to ceftazidime/avibactam, 142/151 (94%) to imipenem/relebactam and 150/151 (99%) to meropenem/vaborbactam. Imipenem/relebactam was the least likely to be active per organism: 46/53 (87%) of *E. coli* isolates, 110/123 (89%) of *K. pneumoniae* isolates and 142/151 (94%) of *E. cloacae* isolates were susceptible. Imipenem/relebactam also had the overall lowest rates of percent susceptibility by culture site, ranging from 35/44 (80%) for respiratory cultures to 76/87 (87%) for sterile sites (Table S2).

**Figure 1.**
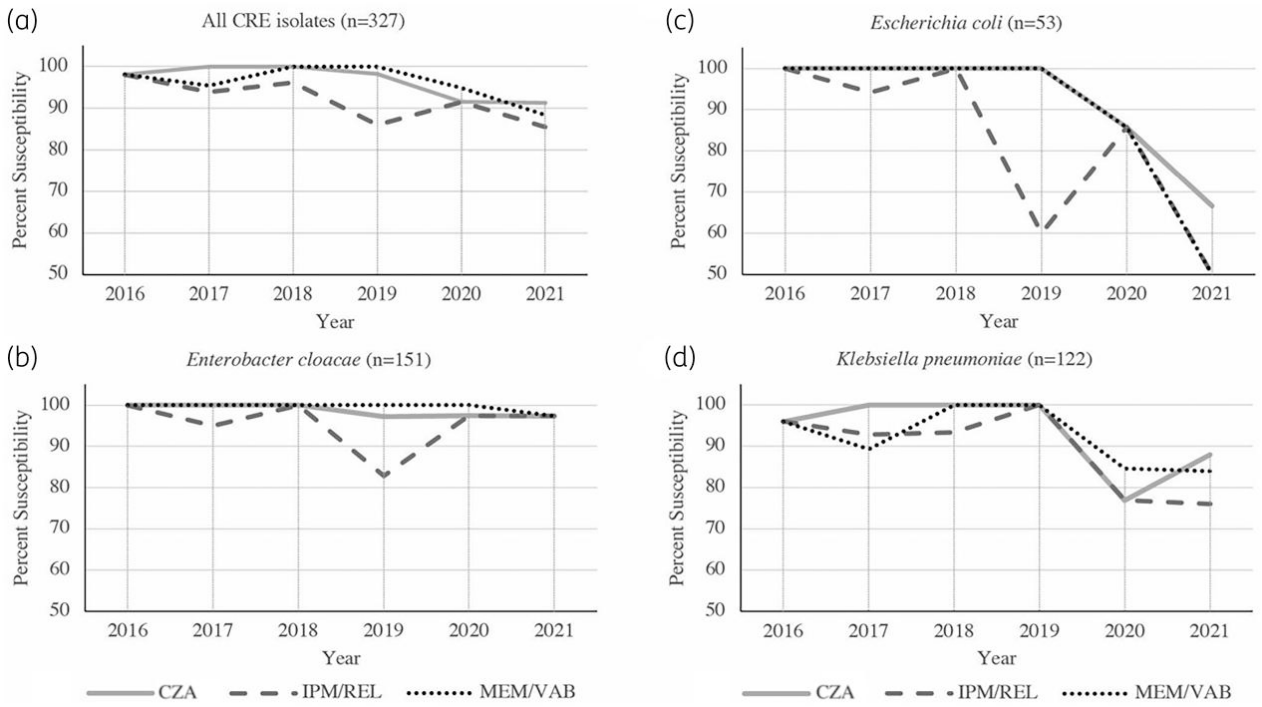
Percent susceptibility of carbapenem-resistant *E. cloacae*, *E. coli* and *K. pneumoniae* isolates to three novel BL/BLI antibiotics, 2016–21. CZA, ceftazidime/avibactam; IPM/REL, imipenem/relebactam; MEM/VAB, meropenem/vaborbactam.

From 2016 to 2021, we observed small incremental decreases in the activity of all three BL/BLIs (Figure 1), with the proportion of susceptible CRE isolates decreasing for ceftazidime/avibactam (98% to 91%), imipenem/relebactam (98% to 86%) and meropenem/vaborbactam (98% to 88%). Of the three organisms, *E. coli* demonstrated the greatest overall decreases in susceptibility to all three BL/BLIs; however, there were fewer than 10 *E. coli* isolates per year from the years 2018–21.

Some CRE isolates susceptible or intermediate to newer BL/BLIs by BMD were found to be HR by PAP. The percent of isolates susceptible/intermediate by BMD but HR by PAP were: 29/314 (9%) for ceftazidime/avibactam, 40/313 (13%) for imipenem/relebactam and 6/311 (2%) for meropenem/vaborbactam. Notably, the highest rates of HR to all three BL/BLIs were among *K. pneumoniae* isolates; 15/116 (13%) susceptible isolates were HR to ceftazidime/avibactam, 30/115 (26%) susceptible/intermediate isolates were HR to imipenem/relebactam and 2/115 (2%) susceptible isolates were HR to meropenem/vaborbactam (Table 2).

**Table 2.** Proportion of CRE isolates susceptible to ceftazidime/avibactam or susceptible/intermediate to imipenem/relebactam and meropenem/vaborbactam by BMD but HR by PAP

CRE isolate species	Susceptible (BMD) *n* (%)	HR (PAP) *n* (%)^a^
Ceftazidime/avibactam		
*E. coli* (*n* = 53)	50 (96)	1 (2)
*K. pneumoniae* (*n* = 123)	116 (94)	15 (13)
*E. cloacae* (*n* = 151)	148 (98)	13 (9)
Total (*n* = 327)	314 (96)	29 (9)
Imipenem/relebactam		
*E. coli* (*n* = 53)	49 (92)	1 (2)
*K. pneumoniae* (*n* = 123)	115 (94)	30 (26)
*E. cloacae* (*n* = 151)	149 (99)	9 (6)
Total (*n* = 327)	313 (96)	40 (13)
Meropenem/vaborbactam		
*E. coli* (*n* = 53)	50 (94)	1 (2)
*K. pneumoniae* (*n* = 123)	115 (93)	2 (2)
*E. cloacae* (*n* = 151)	150 (99)	3 (2)
Total (*n* = 327)	315 (95)	6 (2)

^a^ *n* is the number of CRE isolates testing HR by PAP out of the CRE isolates testing susceptible by BMD. Percentage (%) was calculated by dividing number of CRE isolates HR by PAP by number of CRE isolates susceptible by BMD.

A subset of isolates were HR and/or resistant (HR/R) to different BL/BLIs as determined by PAP, and we therefore grouped these phenotypically non-susceptible isolates together for analysis. In total, 78/327 (24%) CRE isolates were non-susceptible (HR/R) by PAP to at least one BL/BLI (Table S1). Among all CRE, the largest number of isolates, 51/327 (16%), were HR/R to imipenem/relebactam, followed by 42/327 (13%) to ceftazidime/avibactam and 19/327 (6%) to meropenem/vaborbactam. Overall, the proportion of non-susceptible isolates remained low for most of the species–antibiotic combinations, except for *K. pneumoniae* isolates, of which 22/123 (18%) were HR/R to ceftazidime/avibactam and 36/123 (29%) were HR/R to imipenem/relebactam. Non-susceptibility was relatively low and minimally impacted by the HR phenotype for *E. coli* across all antibiotics (Table S3). The percentages of isolates HR/R by culture site are listed in Table S2. The highest rate of susceptibility was found for meropenem/vaborbactam regardless of source, while the highest percentage of HR/R (9/44; 20%) was found for CRE tested against imipenem/relebactam from respiratory cultures.

To begin to understand the mechanisms underlying resistance and HR to these newer BL/BLI, we tested all CRE isolates for (i) phenotypic CP and (ii) identification of Ambler Class A, B or D carbapenemases (Table 3). Out of 327 CRE isolates, 86 (26%) had detectable CP, and among those, 51 (59%) were HR/R to one or more BL/BLI by PAP. Of these 86 CP-CRE, 73 (85%) were identified to have a Class A carbapenemase, 11 (13%) Class B and 2 (2%) Class D. Notably, all Class B CP-CRE isolates were collected in the years 2020 and 2021. Of the 22 isolates that were HR/R to two or all three of the BL/BLIs tested, the majority (17/22; 77%) were CP-CRE and accounted for 9 out of the 11 Class B carbapenemases detected.

**Table 3.** CP by Ambler class in CRE first isolates HR/R, as determined by PAP, to newer BL/BLIs

CRE isolate species	HR/R isolates with any CP *n* (%)	HR/R isolates with Class A carbapenemase *n* (%)^a^	HR/R isolates with Class B carbapenemase *n* (%)^a^	HR/R isolates with Class D carbapenemase *n* (%)^a^
Isolates HR/R to ceftazidime/avibactam				
*E. cloacae* (*n* = 16)	9 (56)	6 (67)	2 (22)	1 (11)
*E. coli* (*n* = 4)	2 (50)	0 (0)	2 (100)	0 (0)
*K. pneumoniae* (*n* = 22)	16 (73)	10 (63)	6 (37)	0 (0)
Total (*n* = 42)	27 (64)	16 (59)	10 (37)	1 (4)
Isolates HR/R to imipenem/relebactam				
*E. cloacae* (*n* = 10)	5 (50)	4 (80)	0 (0)	1 (20)
*E.coli* (*n* = 5)	3 (60)	1 (33)	2 (67)	0 (0)
*K. pneumoniae* (*n* = 37)	29 (78)	22 (76)	6 (21)	1 (4)
Total (*n* = 52)	37 (73)	27 (73)	8 (22)	2 (5)
Isolates HR/R to meropenem/vaborbactam				
*E. cloacae* (*n* = 4)	3 (75)	1 (33)	1 (33)	1 (33)
*E.coli* (*n* = 4)	2 (50)	0 (0)	2 (50)	0 (0)
*K. pneumoniae* (*n* = 11)	9 (82)	3 (33)	6 (67)	0 (0)
Total (*n* = 19)	14 (74)	4 (29)	9 (64)	1 (7)

^a^Percentage (%) calculated by dividing number of CRE isolates HR/R by PAP with the specified Ambler class carbapenemase by total number of HR/R isolates with any CP.

Excluding CRE isolates that tested resistant by PAP, 72 CRE isolates were HR to any BL/BLI by PAP. Of these, 46 (64%) had detectable CP: 39 (50%) were Class A, 5 (13%) were Class B, 2 (2%) were Class D and 26 (35%) were negative for CP. Eighty percent (37/46) of these HR CP-CRE tested susceptible by BMD to all three BL/BLIs; 35/37 (95%) were Ambler Class A CP-CRE, with 1 isolate each for Classes B and D. Table S1 provides the full list of CRE clinical isolates included in this study, with year collected, source, species, BMD results with MIC interpretation and PAP results per BL/BLI tested, and Carba NP results.

## Discussion

From 2016 to 2021, in two academic hospitals in Atlanta, GA, 24% of clinical CRE isolates tested by PAP were resistant or HR to at least one BL/BLI—ceftazidime/avibactam, imipenem/relebactam or meropenem/vaborbactam—despite 90% of these clinical CRE isolates testing susceptible to all three by BMD. The most active BL/BLI against CRE overall was ceftazidime/avibactam. We also observed a 7% to 13% decrease in percent susceptibility to the three BL/BLIs tested over 6 years for CRE first isolates. This may reflect increased antibiotic usage or the introduction of antibiotic resistance genes and changing landscape of carbapenemases in the Southeast.

Recently published work from 74 US medical centres from 2019 to 2021 also found decreases in CRE susceptibility over 3 years: meropenem/vaborbactam decreased from 91.7% to 76.5%, and 92.5% to 78.6% for ceftazidime/avibactam. Most (83%) of this study’s CRE isolates were CP, including KPC (Ambler class A, 65.5%), NDM (Ambler class B, 11%) and OXA-48-like (Ambler class D, 4.6%).^6^ We report a smaller overall percentage of CP-CRE (26%), but the percentages of Ambler class A, B and D carbapenemases are roughly similar to our reported values of 85%, 13% and 2%, respectively.

The high susceptibility of non-CP CRE to the BL/BLIs in this study is not a new finding. Previous work by Tamma *et al.*^4^ has reported high susceptibility rates of non-CP-CRE to the same three BL/BLIs: 87%–93% for *E. coli*, 87%–98% for *K. pneumoniae* and 88%–98% for *E. cloacae* complex. In a study by Castanheira *et al.*,^17^ ceftazidime/avibactam inhibited all non-CP-CREs isolates, while imipenem/relebactam and meropenem/vaborbactam inhibited 93%, and in another study, susceptibility of meropenem/vaborbactam to non-CP-CREs was 97.6%.^18^ It is well documented that resistance to carbapenems may be the result of several mechanisms in addition to the production of carbapenemases, including the production of ESBL and/or AmpC β-lactamase enzymes in association with alteration in outer membrane porins (OMPs).^19–21^ The addition of a BLI in these combination antibiotics may be acting on BLs other than carbapenemases as this new generation of inhibitors—avibactam, relebactam and vaborbactam—are usually active against acquired and intrinsic β-lactamases. The resistance mechanisms among CRE isolates that did not produce carbapenemases are complex and still being deciphered. How BL/BLI combinations work in non-CP-CREs may depend on the bacterial species and their resistance mechanisms.

The presence of HR was observed in all pathogen–antibiotic combinations tested, with rates as high as 26% for *K. pneumoniae* against imipenem/relebactam. Rates of HR were generally the lowest for meropenem/vaborbactam for all three organisms. HR would have been missed not only in CRE isolates that tested susceptible by BMD, but in several that tested resistant by BMD but were HR by PAP. For these isolates, the majority of the population was above the resistant MIC by BMD. However, when PAP is performed the entire population is surveyed and the minority subpopulation that is below the resistant MIC can be calculated.

One of the most common mechanisms of HR described is duplication or amplification of genes encoding antibiotic targets or antibiotic-modifying or -degrading resistance genes or efflux pumps.^8,22–24^ A unique aspect of our study was pairing HR assessment with a phenotypic carbapenemase screen. While CP was detected in the majority of CRE isolates that were HR/R to one or more of the BL/BLIs tested, there remained a consistent subgroup of HR/R isolates whose reduced susceptibility could not be solely attributed to CP and was likely mediated via additional resistance mechanisms. Targeted sequencing and whole-genome sequencing is ongoing to detect other possible mechanisms of HR and resistance, particularly for isolates lacking detectable CP where duplication or amplification of other, non-CP β-lactamases may play a role.

It is unknown if the presence of *in vitro* HR has an impact on clinical outcomes.^8^ There is conflicting evidence that HR can lead to antibiotic treatment failures, and most of these data come from studies of HR in *Staphylococcus aureus*^25–27^ and *Acinetobacter baumannii.*^28–30^ Furthermore, the lack of standardization of HR definitions and methods of detection, especially for Enterobacterales, has made understanding the relevance of HR in treatment failure problematic.^31^ While there are limited data on the clinical implications of HR, animal studies have demonstrated failure of carbapenem therapy in HR Gram-negative bacilli infection models.^32–34^ Future research includes determining if patient- or infection-specific characteristics can predict HR to BL/BLIs, and if HR is associated with poor clinical outcomes.

This study has several limitations. First, the rates of HR-CRE may be underestimated. Despite currently being considered the most reliable standard for detecting HR, PAPs may not be able to capture transient HR, particularly in cases of spontaneous unstable gene amplification.^23,35^ In addition, in this study we excluded repeat isolates from the same patient; however, HR may be more likely to be detected in these patients exposed to many days of antibiotic treatment during their hospital course.^36,37^ Second, we did not have antibiotic usage data available, but an important next step would be to evaluate if the decline in susceptibility to the newer BL/BLIs is associated with increased use of these antibiotics. Third, our study had modest sample sizes of CRE isolates assessed per year per organism. Finally, our data were from two academic hospitals in Atlanta, GA, which may not be generalizable to different hospital systems and regions, as rates of CRE can vary widely by geographical location.^38^

In conclusion, while newer BL/BLIs remain highly active against most CRE, the observed decreases in susceptibility over time suggest a need for ongoing antibiotic stewardship efforts and continued research in the mechanisms of resistance to newer BL/BLIs. HR to BL/BLIs may also be unrecognized in a substantial proportion of CRE isolates. Our work underscores the importance of understanding the local antibiogram when using newer BL/BLIs to treat CRE and that further research is needed to determine how HR should impact treatment decisions.

## Supplementary Material

dlae048_Supplementary_Data

## Supplementary data

Tables S1 to S3 are available as Supplementary data at *JAC-AMR* Online.
