# Supplementary material for: Rates of resistance and heteroresistance to newer β-lactam/β-lactamase inhibitors for carbapenem-resistant Enterobacterales
Source: JAC Antimicrob Resist. 2024 Mar 21;6(2):dlae048. doi: 10.1093/jacamr/dlae048 (PMC10957161; doi:10.1093/jacamr/dlae048)
Supplement: dlae048_Supplementary_Data [file dlae048_supplementary_data.docx]

Supplementary Tables

Table S1: Carbapenem resistant Enterobacterales clinical isolates (n=327) by year collected; source; species; and results of broth microdilution, population analysis profile, and Carba NP testing

|  |  |  |  | Ceftazidime-Avibactam | | | Imipenem-Relebactam | | | Meropenem-Vaborbactam | | |  |
| --- | --- | --- | --- | --- | --- | --- | --- | --- | --- | --- | --- | --- | --- |
| Strain ID | Year | SOURCE | SPECIES | BMD MIC | AST Int | PAP | BMD MIC | AST Int | PAP | BMD MIC | AST Int | PAP | Carba NP Result |
| EC1319 | 2016 | Urine | E. coli | 1 | S | S | 0.125 | S | S | 0.125 | S | S | Neg |
| EC1316 | 2016 | Sterile | E. coli | 1 | S | S | 0.125 | S | S | 0.25 | S | S | Neg |
| EC1317 | 2016 | Other-NS | E. coli | 1 | S | S | 0.125 | S | S | 1 | S | S | Neg |
| EC1313 | 2016 | Sterile | E. coli | 2 | S | S | 1 | S | S | 4 | S | S | Neg |
| EC1311 | 2016 | Urine | E. coli | 1 | S | S | 0.125 | S | S | <0.06 | S | HR | Neg |
| EC601 | 2016 | Urine | E. coli | 0.49 | S | S | 0.5 | S | S | <0.06 | S | S | Neg |
| EC1312 | 2016 | Sterile | E. coli | 1 | S | S | <0.06 | S | S | <0.06 | S | S | Class A |
| EC602 | 2016 | Urine | E. coli | 0.49 | S | S | 0.125 | S | S | <0.06 | S | S | Neg |
| EC603 | 2016 | Sterile | E. coli | 1 | S | S | 0.5 | S | S | <0.06 | S | S | Neg |
| EC605 | 2016 | Urine | E. coli | 0.49 | S | S | 0.25 | S | S | <0.06 | S | S | Class A |
| EC1315 | 2016 | Urine | E. coli | 0.25 | S | S | 0.125 | S | S | <0.06 | S | S | Neg |
| EC1318 | 2016 | Sterile | E. coli | 0.25 | S | S | 0.125 | S | S | <0.06 | S | S | Neg |
| EC1321 | 2016 | Urine | E. coli | 1 | S | S | 0.25 | S | S | <0.06 | S | S | Neg |
| EC1337 | 2017 | Urine | E. coli | 2 | S | S | 0.5 | S | S | 0.125 | S | S | Neg |
| EC1330 | 2017 | Respiratory | E. coli | 0.5 | S | S | 0.5 | S | S | 0.25 | S | S | Neg |
| EC1331 | 2017 | Urine | E. coli | 2 | S | S | 0.125 | S | S | 0.5 | S | S | Neg |
| EC1322 | 2017 | Other-NS | E. coli | 4 | S | S | 0.25 | S | S | 1 | S | S | Neg |
| EC1323 | 2017 | Sterile | E. coli | 2 | S | S | 4 | R | HR | 4 | S | S | Class A |
| EC1325 | 2017 | Other-NS | E. coli | 0.5 | S | S | 0.125 | S | S | <0.06 | S | S | Neg |
| EC700 | 2017 | Urine | E. coli | 0.49 | S | S | 0.25 | S | S | <0.06 | S | S | Neg |
| EC701 | 2017 | Urine | E. coli | 0.49 | S | S | 0.5 | S | S | <0.06 | S | S | Neg |
| EC702 | 2017 | Urine | E. coli | 0.49 | S | S | 0.125 | S | S | <0.06 | S | S | Neg |
| EC1328 | 2017 | Urine | E. coli | 2 | S | S | 0.125 | S | S | <0.06 | S | S | Neg |
| EC1329 | 2017 | Urine | E. coli | <0.06 | S | S | 1 | S | S | <0.06 | S | S | Neg |
| EC704 | 2017 | Urine | E. coli | 0.49 | S | S | 0.5 | S | S | <0.06 | S | S | Neg |
| EC1332 | 2017 | Sterile | E. coli | 0.5 | S | S | 0.25 | S | S | <0.06 | S | S | Neg |
| EC1333 | 2017 | Urine | E. coli | 0.5 | S | S | 0.5 | S | S | <0.06 | S | S | Neg |
| EC706 | 2017 | Urine | E. coli | 0.49 | S | S | 0.25 | S | S | <0.06 | S | S | Neg |
| EC1336 | 2017 | Sterile | E. coli | 1 | S | S | 0.125 | S | S | <0.06 | S | S | Class A |
| EC707 | 2017 | Urine | E. coli | 0.49 | S | S | 0.25 | S | S | <0.06 | S | S | Neg |
| EC800 | 2018 | Urine | E. coli | 0.49 | S | S | 0.5 | S | S | <0.06 | S | S | Neg |
| EC1338 | 2018 | Sterile | E. coli | 2 | S | S | 0.125 | S | S | <0.06 | S | S | Neg |
| EC803 | 2018 | Sterile | E. coli | 4 | S | S | 0.25 | S | S | <0.06 | S | S | Neg |
| EC1339 | 2018 | Urine | E. coli | 1 | S | S | 0.125 | S | S | <0.06 | S | S | Neg |
| EC1340 | 2018 | Respiratory | E. coli | 2 | S | S | 0.125 | S | S | <0.06 | S | S | Neg |
| EC203 | 2019 | Sterile | E. coli | 0.49 | S | S | 0.125 | S | S | 0.49 | S | S | Neg |
| EC430 | 2019 | Urine | E. coli | 0.5 | S | S | 0.25 | S | S | 0.5 | S | S | Neg |
| EC403 | 2019 | Other-NS | E. coli | 0.25 | S | S | 1 | S | S | 1 | S | S | Neg |
| EC439 | 2019 | Sterile | E. coli | 0.125 | S | S | 2 | I | HR | 1 | S | S | Neg |
| EC437 | 2019 | Other-NS | E. coli | 2 | S | HR | 2 | I | S | 4 | S | S | Neg |
| EC573 | 2020 | Urine | E. coli | 0.06 | S | S | 1 | S | S | 0.125 | S | S | Neg |
| EC568 | 2020 | Urine | E. coli | 0.49 | S | S | 0.249 | S | S | 0.49 | S | S | Neg |
| EC1342 | 2020 | Other-NS | E. coli | 2 | S | S | 0.25 | S | S | 0.5 | S | S | Neg |
| EC567 | 2020 | Urine | E. coli | 17 | R | R | 16 | R | HR | 17 | R | HR | Class B |
| EC569 | 2020 | Urine | E. coli | 0.5 | S | S | 0.25 | S | S | <0.06 | S | S | Neg |
| EC531 | 2020 | Sterile | E. coli | 0.5 | S | S | 0.5 | S | S | <0.06 | S | S | Neg |
| EC1348 | 2020 | Respiratory | E. coli | 0.125 | S | S | 0.25 | S | S | <0.06 | S | S | Neg |
| EC1302 | 2021 | Urine | E. coli | 4 | S | S | 1 | S | S | 4 | S | S | Neg |
| EC1305 | 2021 | Urine | E. coli | 2 | S | S | 2 | I | S | 12 | I | S | Neg |
| EC1304 | 2021 | Urine | E. coli | <0.06 | S | S | 0.125 | S | S | <0.06 | S | S | Neg |
| EC1303 | 2021 | Urine | E. coli | <0.06 | S | S | 0.125 | S | S | <0.06 | S | S | Neg |
| EC1306 | 2021 | Urine | E. coli | >32 | R | R | 16 | R | HR | >32 | R | HR | Neg |
| EC1301 | 2021 | Urine | E. coli | >32 | R | R | >32 | R | HR | >32 | R | HR | Class B |
| K600 | 2016 | Urine | KPN | 1 | S | S | 1 | S | HR | 0.125 | S | S | Class A |
| K990 | 2016 | Sterile | KPN | 2 | S | S | 0.5 | S | S | 0.125 | S | S | Class A |
| K601 | 2016 | Urine | KPN | 0.49 | S | S | 0.25 | S | S | <0.06 | S | HR | Class A |
| K602 | 2016 | Urine | KPN | 4 | S | HR | 1 | S | HR | 1 | S | HR | Class A |
| K991 | 2016 | Respiratory | KPN | 4 | S | S | 0.5 | S | HR | 2 | S | S | Class A |
| K993 | 2016 | Urine | KPN | 1 | S | S | 0.5 | S | S | <0.06 | S | S | Class A |
| K604 | 2016 | Urine | KPN | 1 | S | S | 1 | S | HR | 0.125 | S | S | Class A |
| K995 | 2016 | Sterile | KPN | 2 | S | S | 0.25 | S | S | <0.06 | S | S | Class A |
| K996 | 2016 | Sterile | KPN | 2 | S | HR | 0.5 | S | S | <0.06 | S | S | Neg |
| K999 | 2016 | Urine | KPN | 2 | S | S | 0.125 | S | S | 0.125 | S | S | Neg |
| K1000 | 2016 | Urine | KPN | 1 | S | S | 1 | S | HR | <0.06 | S | S | Class A |
| K607 | 2016 | Urine | KPN | 4 | S | HR | 1 | S | HR | 4 | S | S | Class A |
| K608 | 2016 | Urine | KPN | 17 | R | R | >32 | R | R | >32 | R | R | Neg |
| K1001 | 2016 | Urine | KPN | 0.5 | S | S | 0.25 | S | S | 0.25 | S | S | Neg |
| K1002 | 2016 | Respiratory | KPN | 0.5 | S | S | 0.125 | S | S | <0.06 | S | S | Neg |
| K1006 | 2016 | Urine | KPN | 1 | S | S | 0.5 | S | S | 0.25 | S | S | Class A |
| K611 | 2016 | Urine | KPN | 0.49 | S | S | 0.125 | S | S | <0.06 | S | S | N |
| K1009 | 2016 | Respiratory | KPN | 1 | S | S | 1 | S | HR | <0.06 | S | S | Class A |
| K1010 | 2016 | Respiratory | KPN | 1 | S | S | 0.5 | S | S | <0.06 | S | S | Neg |
| K1011 | 2016 | Sterile | KPN | 2 | S | S | 1 | S | HR | 0.5 | S | S | Neg |
| K1012 | 2016 | Sterile | KPN | 1 | S | S | 0.25 | S | S | 0.5 | S | S | Neg |
| K1013 | 2016 | Urine | KPN | 1 | S | S | 1 | S | HR | <0.06 | S | S | Class A |
| K1014 | 2016 | Other-NS | KPN | 0.5 | S | S | 0.5 | S | HR | <0.06 | S | S | Class A |
| K1017 | 2016 | Urine | KPN | 2 | S | S | 0.25 | S | HR | 0.125 | S | S | Class A |
| K1019 | 2016 | Sterile | KPN | 2 | S | S | 0.5 | S | S | 4 | S | S | Neg |
| K700 | 2017 | Urine | KPN | 1 | S | S | 1 | S | HR | <0.06 | S | S | Class A |
| K702 | 2017 | Urine | KPN | 1 | S | S | 0.25 | S | S | <0.06 | S | S | Class A |
| K703 | 2017 | Urine | KPN | 1 | S | S | 1 | S | S | 1 | S | S | Neg |
| K704 | 2017 | Urine | KPN | 0.49 | S | S | 2 | I | S | <0.06 | S | S | Neg |
| K1024 | 2017 | Respiratory | KPN | 2 | S | S | 0.25 | S | HR | 1 | S | R | Neg |
| K1025 | 2017 | Urine | KPN | 2 | S | S | 0.25 | S | HR | <0.06 | S | S | Class A |
| K1026 | 2017 | Urine | KPN | 2 | S | S | 0.125 | S | HR | <0.06 | S | S | Class A |
| K1027 | 2017 | Sterile | KPN | 2 | S | S | 0.25 | S | S | 2 | S | S | Neg |
| K1028 | 2017 | Urine | KPN | 4 | S | HR | 0.25 | S | HR | 4 | S | S | Class A |
| K1031 | 2017 | Sterile | KPN | 0.25 | S | S | 0.125 | S | S | <0.06 | S | S | Neg |
| K705 | 2017 | Urine | KPN | 0.49 | S | S | 0.5 | S | S | <0.06 | S | S | Class A |
| K1032 | 2017 | Urine | KPN | 4 | S | S | 2 | I | S | 0.5 | S | S | Class A |
| K1033 | 2017 | Urine | KPN | 4 | S | HR | 1 | S | HR | 8 | I | S | Neg |
| K706 | 2017 | Sterile | KPN | 0.49 | S | S | 0.25 | S | S | <0.06 | S | S | Neg |
| K707 | 2017 | Urine | KPN | 0.49 | S | S | 0.125 | S | S | <0.06 | S | S | Neg |
| K1035 | 2017 | Urine | KPN | 2 | S | S | 1 | S | S | 8 | I | S | Neg |
| K1037 | 2017 | Sterile | KPN | 2 | S | S | 0.25 | S | HR | <0.06 | S | S | Class A |
| K708 | 2017 | Urine | KPN | 1 | S | S | 0.5 | S | S | <0.06 | S | S | Class A |
| K1038 | 2017 | Respiratory | KPN | 2 | S | S | 0.25 | S | S | 0.5 | S | S | Neg |
| K1039 | 2017 | Respiratory | KPN | 2 | S | S | 1 | S | HR | 8 | I | S | Class A |
| K709 | 2017 | Urine | KPN | 4 | S | S | 1 | S | S | <0.06 | S | S | Class A |
| K1042 | 2017 | Urine | KPN | 2 | S | S | 1 | S | HR | 0.5 | S | S | Class A |
| K1043 | 2017 | Respiratory | KPN | 0.5 | S | S | 0.5 | S | S | <0.06 | S | S | Neg |
| K1046 | 2017 | Urine | KPN | 2 | S | S | 0.5 | S | S | 0.125 | S | S | Neg |
| K1047 | 2017 | Other-NS | KPN | 4 | S | S | 1 | S | HR | 1 | S | S | Class A |
| K710 | 2017 | Sterile | KPN | 8 | S | S | 0.25 | S | S | 0.5 | S | S | Neg |
| K1048 | 2017 | Respiratory | KPN | 2 | S | HR | 0.5 | S | HR | 4 | S | S | Neg |
| K711 | 2017 | Urine | KPN | 1 | S | S | 1 | S | HR | 0.25 | S | S | Class A |
| K1051 | 2018 | Sterile | KPN | <0.06 | S | S | <0.06 | S | S | 0.25 | S | S | Neg |
| K800 | 2018 | Urine | KPN | 1 | S | S | 1 | S | S | 0.49 | S | S | Class A |
| K1053 | 2018 | Urine | KPN | 4 | S | S | 1 | S | S | 0.5 | S | S | Neg |
| K802 | 2018 | Urine | KPN | 0.49 | S | S | 1 | S | HR | 0.5 | S | S | Class D |
| K803 | 2018 | Urine | KPN | 0.49 | S | S | 0.5 | S | S | 0.125 | S | S | Class A |
| K804 | 2018 | Urine | KPN | 1 | S | S | 0.125 | S | S | 0.49 | S | S | Neg |
| K1055 | 2018 | Sterile | KPN | 4 | S | HR | 0.25 | S | S | 0.125 | S | S | Class A |
| K806 | 2018 | Urine | KPN | 0.49 | S | S | 1 | S | S | 0.25 | S | S | Neg |
| K1058 | 2018 | Urine | KPN | 1 | S | S | 0.5 | S | S | 0.125 | S | S | Neg |
| K807 | 2018 | Urine | KPN | 1 | S | S | 0.25 | S | HR | <0.06 | S | S | Class A |
| K808 | 2018 | Urine | KPN | 0.49 | S | S | 2 | I | S | 0.49 | S | S | Neg |
| K1060 | 2018 | Respiratory | KPN | 1 | S | S | 0.125 | S | S | 0.25 | S | S | Neg |
| K810 | 2018 | Sterile | KPN | 0.49 | S | S | 1 | S | S | 0.49 | S | S | Class A |
| K1061 | 2018 | Sterile | KPN | 2 | S | S | 0.5 | S | HR | 0.5 | S | S | Neg |
| K1062 | 2018 | Urine | KPN | 0.5 | S | S | 0.5 | S | S | <0.06 | S | S | Neg |
| K259 | 2019 | Urine | KPN | 1 | S | S | 0.25 | S | S | 0.5 | S | S | Neg |
| K1063 | 2019 | Sterile | KPN | 1 | S | S | 1 | S | S | 0.5 | S | S | Neg |
| K1065 | 2019 | Other-NS | KPN | 0.5 | S | S | 0.25 | S | S | 0.5 | S | S | Neg |
| K152 | 2019 | Urine | KPN | 1 | S | S | 0.249 | S | S | 0.49 | S | S | Neg |
| K280 | 2019 | Sterile | KPN | 2 | S | S | 0.5 | S | S | 0.5 | S | S | Neg |
| K171 | 2019 | Urine | KPN | 0.49 | S | S | 0.25 | S | S | 0.49 | S | S | Neg |
| K1066 | 2019 | Respiratory | KPN | 2 | S | S | 0.125 | S | S | <0.06 | S | S | Class A |
| K1 | 2019 | Sterile | KPN | 0.25 | S | S | 0.125 | S | S | 2 | S | S | Neg |
| K293 | 2019 | Other-NS | KPN | 2 | S | S | 0.25 | S | S | 0.5 | S | S | Neg |
| K1067 | 2019 | Respiratory | KPN | 8 | S | S | 0.25 | S | S | <0.06 | S | S | Class A |
| K2 | 2019 | Sterile | KPN | 1 | S | S | 0.5 | S | HR | <0.06 | S | S | Neg |
| K3 | 2019 | Sterile | KPN | 2 | S | S | 0.249 | S | S | 1 | S | S | Neg |
| K1068 | 2019 | Respiratory | KPN | 4 | S | S | 1 | S | S | 1 | S | S | Class A |
| K4 | 2019 | Sterile | KPN | 1 | S | HR | 0.125 | S | S | 2 | S | S | Neg |
| K303 | 2019 | Other-NS | KPN | 2 | S | S | 1 | S | S | 2 | S | S | Neg |
| K1069 | 2019 | Respiratory | KPN | 2 | S | S | 1 | S | HR | <0.06 | S | S | Class A |
| K318 | 2019 | Sterile | KPN | 0.5 | S | S | 0.125 | S | S | 0.5 | S | S | Neg |
| K501 | 2020 | Sterile | KPN | 1 | S | S | 1 | S | S | >0.06 | S | S | Class A |
| K555 | 2020 | Urine | KPN | 0.5 | S | S | 1 | S | S | 0.25 | S | S | Neg |
| K556 | 2020 | Urine | KPN | 2 | S | HR | 0.5 | S | S | 0.49 | S | S | Class A |
| K557 | 2020 | Urine | KPN | 0.5 | S | HR | 0.125 | S | S | <0.06 | S | S | Class A |
| K559 | 2020 | Respiratory | KPN | 4 | S | S | 1 | S | S | 1 | S | S | Neg |
| K560 | 2020 | Urine | KPN | 4 | S | S | 0.125 | S | HR | 0.5 | S | S | Neg |
| K562 | 2020 | Urine | KPN | 17 | R | R | 17 | R | R | 17 | R | R | Class B |
| K563 | 2020 | Respiratory | KPN | >32 | R | R | >32 | R | R | >32 | R | R | Class B |
| K1072 | 2020 | Sterile | KPN | 4 | S | HR | 0.5 | S | S | 2 | S | S | Neg |
| K1073 | 2020 | Respiratory | KPN | 2 | S | S | 0.25 | S | S | 0.125 | S | S | N |
| K534 | 2020 | Sterile | KPN | 1 | S | S | 1 | S | HR | >0.06 | S | S | Class A |
| K574 | 2020 | Urine | KPN | 4 | S | S | 1 | S | S | 1 | S | S | N |
| K1077 | 2020 | Sterile | KPN | >32 | R | R | >32 | R | R | >32 | R | R | Class B |
| K957 | 2021 | Sterile | KPN | <0.06 | S | HR | 0.125 | S | S | 0.25 | S | S | Class A |
| K981 | 2021 | Other-NS | KPN | 1 | S | S | 2 | I | S | 2 | S | S | N |
| K966 | 2021 | Urine | KPN | 8 | S | HR | 8 | S | S | 2 | R | R | Class A |
| K963 | 2021 | Urine | KPN | 8 | S | S | 0.5 | S | S | <0.06 | S | S | Class A |
| K964 | 2021 | Urine | KPN | 4 | S | S | 0.25 | S | S | <0.06 | S | S | N |
| K982 | 2021 | Urine | KPN | 2 | S | S | 2 | I | S | 0.125 | S | S | Class A |
| K965 | 2021 | Urine | KPN | 4 | S | S | 0.25 | S | S | 0.5 | S | S | N |
| K954 | 2021 | Respiratory | KPN | 0.25 | S | S | 0.25 | S | S | 0.5 | S | S | N |
| K980 | 2021 | Urine | KPN | <0.06 | S | S | 0.5 | S | S | 0.5 | S | S | Class A |
| K962 | 2021 | Urine | KPN | >32 | R | R | >32 | R | R | >32 | R | R | Class B |
| K976 | 2021 | Sterile | KPN | >32 | R | R | >32 | R | R | >32 | R | R | Class B |
| K969 | 2021 | Urine | KPN | 0.5 | S | S | 0.125 | S | S | 0.5 | S | S | N |
| K959 | 2021 | Respiratory | KPN | 0.25 | S | S | 0.5 | S | S | 0.25 | S | S | N |
| K972 | 2021 | Urine | KPN | 0.25 | S | S | 0.25 | S | S | 1 | S | S | N |
| K960 | 2021 | Sterile | KPN | 4 | S | S | 0.25 | S | S | 4 | S | S | Class A |
| K974 | 2021 | Respiratory | KPN | >32 | R | R | >32 | R | R | >32 | R | R | Class B |
| K975 | 2021 | Sterile | KPN | 1 | S | S | 0.25 | S | S | 2 | S | S | N |
| K971 | 2021 | Urine | KPN | <0.06 | S | S | 1 | S | S | 0.5 | S | S | Class A |
| K967 | 2021 | Respiratory | KPN | 2 | S | S | 2 | I | S | 2 | S | S | N |
| K979 | 2021 | Sterile | KPN | 2 | S | S | 0.25 | S | S | 2 | S | S | N |
| K956 | 2021 | Sterile | KPN | 4 | S | S | 0.25 | S | S | <0.06 | S | S | Class A |
| K977 | 2021 | Sterile | KPN | 0.125 | S | HR | 0.125 | S | S | <0.06 | S | S | Class A |
| K978 | 2021 | Sterile | KPN | 0.125 | S | S | 0.125 | S | S | <0.06 | S | S | Class A |
| K968 | 2021 | Respiratory | KPN | <0.06 | S | HR | 0.25 | S | S | <0.06 | S | S | Class A |
| K973 | 2021 | Sterile | KPN | 4 | S | S | 0.25 | S | S | <0.06 | S | S | Class A |
| EB600 | 2016 | Urine | E. cloacae | 1 | S | S | 0.25 | S | S | <0.06 | S | S | Class A |
| EB805 | 2016 | Urine | E. cloacae | 0.25 | S | S | <0.06 | S | S | 0.5 | S | S | N |
| EB806 | 2016 | Urine | E. cloacae | 2 | S | S | 1 | S | S | 0.25 | S | S | N |
| EB602 | 2016 | Urine | E. cloacae | 0.49 | S | S | 0.25 | S | S | <0.06 | S | S | N |
| EB807 | 2016 | Sterile | E. cloacae | 1 | S | S | <0.06 | S | S | <0.06 | S | S | N |
| EB809 | 2016 | Sterile | E. cloacae | 1 | S | HR | 0.125 | S | S | <0.06 | S | S | N |
| EB604 | 2016 | Urine | E. cloacae | 1 | S | S | 0.5 | S | S | <0.06 | S | S | N |
| EB605 | 2016 | Urine | E. cloacae | 0.49 | S | S | 0.25 | S | S | 0.125 | S | S | N |
| EB810 | 2016 | Sterile | E. cloacae | 1 | S | S | 0.125 | S | S | <0.06 | S | S | N |
| EB811 | 2016 | Urine | E. cloacae | 2 | S | S | 0.25 | S | S | 0.5 | S | S | Class A |
| EB812 | 2016 | Sterile | E. cloacae | 2 | S | S | 0.5 | S | S | 0.125 | S | S | N |
| EB606 | 2016 | Urine | E. cloacae | 2 | S | S | 1 | S | S | 0.25 | S | S | N |
| EB813 | 2016 | Respiratory | E. cloacae | 2 | S | S | 0.125 | S | S | 0.25 | S | S | N |
| EB814 | 2017 | Sterile | E. cloacae | 8 | S | HR | 2 | I | HR | 2 | S | S | N |
| EB700 | 2017 | Urine | E. cloacae | 1 | S | S | 0.5 | S | S | 0.25 | S | S | N |
| EB815 | 2017 | Sterile | E. cloacae | 2 | S | S | 0.125 | S | S | 0.125 | S | S | Class A |
| EB816 | 2017 | Respiratory | E. cloacae | 2 | S | S | 0.125 | S | S | <0.06 | S | S | N |
| EB817 | 2017 | Respiratory | E. cloacae | 2 | S | S | <0.06 | S | S | <0.06 | S | S | N |
| EB818 | 2017 | Respiratory | E. cloacae | 2 | S | S | <0.06 | S | S | 0.25 | S | S | N |
| EB703 | 2017 | Urine | E. cloacae | 2 | S | S | 1 | S | S | 0.125 | NA | S | N |
| EB704 | 2017 | Urine | E. cloacae | 2 | S | S | 0.5 | S | S | <0.06 | S | S | N |
| EB819 | 2017 | Sterile | E. cloacae | 2 | S | S | 0.125 | S | S | <0.06 | S | S | N |
| EB820 | 2017 | Sterile | E. cloacae | 2 | S | S | 0.125 | S | S | 0.125 | S | S | N |
| EB821 | 2017 | Respiratory | E. cloacae | 2 | S | S | 0.125 | S | S | 0.125 | S | S | N |
| EB822 | 2017 | Other-NS | E. cloacae | 2 | S | S | 0.5 | S | S | 0.25 | S | S | N |
| EB705 | 2017 | Sterile | E. cloacae | 1 | S | S | 0.25 | S | S | <0.06 | S | S | N |
| EB823 | 2017 | Urine | E. cloacae | 2 | S | S | 0.25 | S | S | 0.25 | S | S | N |
| EB824 | 2017 | Urine | E. cloacae | 2 | S | S | 0.25 | S | S | 0.125 | S | S | N |
| EB826 | 2017 | Urine | E. cloacae | 4 | S | S | 0.25 | S | S | 0.125 | S | S | N |
| EB829 | 2017 | Sterile | E. cloacae | 2 | S | S | 0.25 | S | S | <0.06 | S | S | N |
| EB830 | 2017 | Other-NS | E. cloacae | 1 | S | S | 0.5 | S | S | <0.06 | S | S | N |
| EB831 | 2017 | Other-NS | E. cloacae | 2 | S | HR | 0.25 | S | S | 1 | S | S | N |
| EB832 | 2017 | Respiratory | E. cloacae | 2 | S | S | 0.125 | S | S | <0.06 | S | S | N |
| EB802 | 2018 | Sterile | E. cloacae | 8 | S | HR | 0.25 | S | S | 0.125 | S | S | Class A |
| EB834 | 2018 | Other-NS | E. cloacae | 2 | S | S | 0.25 | S | S | <0.06 | S | S | N |
| EB803 | 2018 | Sterile | E. cloacae | 8 | S | S | 0.5 | S | S | <0.06 | S | S | N |
| EB800 | 2018 | Sterile | E. cloacae | 0.5 | S | S | 0.5 | S | S | <0.06 | S | S | Class A |
| EB836 | 2018 | Other-NS | E. cloacae | 2 | S | S | 0.25 | S | S | <0.06 | S | S | N |
| EB804 | 2018 | Urine | E. cloacae | 2 | S | HR | 0.5 | S | S | 1 | S | S | N |
| EB4 | 2019 | Sterile | E. cloacae | 2 | S | HR | 0.5 | S | S | 0.5 | S | S | Class A |
| EB16 | 2019 | Sterile | E. cloacae | 2 | S | S | 2 | I | S | <0.06 | S | S | N |
| EB17 | 2019 | Sterile | E. cloacae | 0.49 | S | S | 0.49 | S | S | 0.49 | S | S | N |
| EB18 | 2019 | Urine | E. cloacae | 8 | S | S | 1 | S | S | <0.06 | S | S | N |
| EB19 | 2019 | Urine | E. cloacae | 2 | S | S | 2 | I | S | <0.06 | S | S | N |
| EB20 | 2019 | Sterile | E. cloacae | 4 | S | HR | 1 | S | S | 0.49 | S | S | N |
| EB837 | 2019 | Other-NS | E. cloacae | 2 | S | S | 0.25 | S | S | 0.125 | S | S | N |
| EB24 | 2019 | Urine | E. cloacae | 1 | S | S | 1 | S | S | <0.06 | S | S | N |
| EB25 | 2019 | Other-NS | E. cloacae | 2 | S | S | 2 | I | S | <0.06 | S | S | N |
| EB838 | 2019 | Other-NS | E. cloacae | 2 | S | S | 0.5 | S | S | <0.06 | S | S | N |
| EB27 | 2019 | Urine | E. cloacae | 1 | S | S | 1 | S | S | 0.125 | S | S | N |
| EB28 | 2019 | Sterile | E. cloacae | 4 | S | HR | 0.25 | S | S | <0.06 | S | S | Class A |
| EB29 | 2019 | Other-NS | E. cloacae | 1 | S | S | 1 | S | S | <0.06 | S | S | N |
| EB30 | 2019 | Urine | E. cloacae | 1 | S | S | 1 | S | S | <0.06 | S | S | N |
| EB839 | 2019 | Other-NS | E. cloacae | 16 | R | R | 0.25 | S | HR | 1 | S | S | Class A |
| EB32 | 2019 | Urine | E. cloacae | 2 | S | S | 2 | I | S | <0.06 | S | S | N |
| EB33 | 2019 | Other-NS | E. cloacae | 1 | S | S | 1 | S | S | <0.06 | S | S | N |
| EB34 | 2019 | Urine | E. cloacae | 8 | S | HR | 0.49 | S | HR | 0.49 | S | S | Class A |
| EB35 | 2019 | Sterile | E. cloacae | 1 | S | S | 1 | S | S | <0.06 | S | S | N |
| EB36 | 2019 | Urine | E. cloacae | 1 | S | S | 0.49 | S | S | 0.49 | S | S | N |
| EB37 | 2019 | Urine | E. cloacae | 2 | S | S | 0.49 | S | S | 0.49 | S | S | N |
| EB38 | 2019 | Other-NS | E. cloacae | 1 | S | HR | 1 | S | S | <0.06 | S | S | Class A |
| EB841 | 2019 | Respiratory | E. cloacae | 2 | S | S | 0.125 | S | S | <0.06 | S | S | N |
| EB40 | 2019 | Urine | E. cloacae | 0.5 | S | S | 0.5 | S | S | <0.06 | S | S | N |
| EB41 | 2019 | Urine | E. cloacae | 1 | S | S | 0.49 | S | S | 0.49 | S | S | N |
| EB42 | 2019 | Urine | E. cloacae | 2 | S | S | 2 | I | S | 0.125 | S | S | N |
| EB43 | 2019 | Urine | E. cloacae | 0.49 | S | S | 0.49 | S | S | 0.49 | S | S | N |
| EB44 | 2019 | Other-NS | E. cloacae | 1 | S | S | 1 | S | S | 0.125 | S | S | N |
| EB48 | 2019 | Urine | E. cloacae | 2 | S | S | 0.49 | S | S | 0.49 | S | S | N |
| EB842 | 2019 | Sterile | E. cloacae | 2 | S | S | 0.125 | S | S | 0.5 | S | S | N |
| EB49 | 2019 | Urine | E. cloacae | 1 | S | S | 1 | S | S | <0.06 | S | S | N |
| EB50 | 2019 | Sterile | E. cloacae | 1 | S | S | 0.49 | S | S | 0.49 | S | S | N |
| EB51 | 2019 | Urine | E. cloacae | 1 | S | S | 1 | S | S | <0.06 | S | S | N |
| EB52 | 2019 | Sterile | E. cloacae | 1 | S | S | 1 | S | S | <0.06 | S | S | N |
| EB53 | 2019 | Urine | E. cloacae | 2 | S | S | 2 | I | S | 0.125 | S | S | N |
| EB515 | 2020 | Urine | E. cloacae | 2 | S | S | 0.249 | S | S | 0.49 | S | S | N |
| EB843 | 2020 | Sterile | E. cloacae | 4 | S | HR | 0.25 | S | S | 0.125 | S | S | N |
| EB844 | 2020 | Sterile | E. cloacae | 2 | S | S | 0.25 | S | S | <0.06 | S | S | N |
| EB845 | 2020 | Urine | E. cloacae | 4 | S | S | 0.125 | S | S | <0.06 | S | S | N |
| EB846 | 2020 | Other-NS | E. cloacae | 4 | S | S | 0.25 | S | S | 0.125 | S | S | N |
| EB847 | 2020 | Respiratory | E. cloacae | 4 | S | S | 0.25 | S | S | <0.06 | S | S | N |
| EB848 | 2020 | Sterile | E. cloacae | 2 | S | S | 0.25 | S | S | <0.06 | S | S | N |
| EB517 | 2020 | Urine | E. cloacae | 4 | S | S | 0.249 | S | S | 0.49 | S | S | N |
| EB503 | 2020 | Sterile | E. cloacae | 1 | S | S | 0.249 | S | S | 0.49 | S | S | N |
| EB519 | 2020 | Urine | E. cloacae | 4 | S | S | 0.249 | S | S | 0.49 | S | S | N |
| EB520 | 2020 | Urine | E. cloacae | 1 | S | S | 0.5 | S | S | <0.06 | S | S | N |
| EB850 | 2020 | Respiratory | E. cloacae | 4 | S | S | 0.25 | S | HR | <0.06 | S | S | Class A |
| EB851 | 2020 | Other-NS | E. cloacae | 2 | S | S | 0.25 | S | S | <0.06 | S | S | N |
| EB852 | 2020 | Respiratory | E. cloacae | 2 | S | S | 0.25 | S | S | <0.06 | S | S | N |
| EB505 | 2020 | Sterile | E. cloacae | 0.49 | S | S | 0.249 | S | S | 0.49 | S | S | N |
| EB521 | 2020 | Sterile | E. cloacae | 2 | S | S | 0.25 | S | S | 0.125 | S | S | N |
| EB522 | 2020 | Urine | E. cloacae | 0.49 | S | S | 0.249 | S | S | 0.49 | S | S | N |
| EB523 | 2020 | Urine | E. cloacae | 1 | S | S | 0.249 | S | S | 0.49 | S | S | N |
| EB524 | 2020 | Urine | E. cloacae | 8 | S | S | 0.25 | S | S | 0.25 | S | S | N |
| EB855 | 2020 | Respiratory | E. cloacae | 4 | S | S | 0.125 | S | S | <0.06 | S | S | N |
| EB525 | 2020 | Urine | E. cloacae | 2 | S | S | 0.5 | S | S | 0.125 | S | S | N |
| EB856 | 2020 | Other-NS | E. cloacae | 2 | S | HR | 0.125 | S | S | <0.06 | S | S | Class B |
| EB526 | 2020 | Urine | E. cloacae | 0.49 | S | S | 0.249 | S | S | 0.49 | S | S | N |
| EB527 | 2020 | Other-NS | E. cloacae | 1 | S | S | 0.5 | S | HR | 0.25 | S | S | N |
| EB528 | 2020 | Other-NS | E. cloacae | 2 | S | S | 0.25 | S | S | 0.125 | S | S | N |
| EB530 | 2020 | Urine | E. cloacae | 8 | S | S | 0.25 | S | S | <0.06 | S | S | N |
| EB531 | 2020 | Other-NS | E. cloacae | 1 | S | S | 0.5 | S | S | <0.06 | S | S | N |
| EB532 | 2020 | Urine | E. cloacae | 2 | S | S | 0.5 | S | S | <0.06 | S | S | N |
| EB533 | 2020 | Urine | E. cloacae | 2 | S | S | 0.25 | S | S | <0.06 | S | S | N |
| EB857 | 2020 | Respiratory | E. cloacae | 4 | S | S | 0.125 | S | S | <0.06 | S | S | N |
| EB536 | 2020 | Urine | E. cloacae | 2 | S | S | 0.125 | S | S | 0.125 | S | S | N |
| EB537 | 2020 | Sterile | E. cloacae | 1 | S | S | 0.125 | S | S | 0.25 | S | S | N |
| EB858 | 2020 | Other-NS | E. cloacae | 4 | S | S | 0.25 | S | S | 0.5 | S | S | N |
| EB860 | 2020 | Other-NS | E. cloacae | 8 | S | S | 0.25 | S | HR | 0.125 | S | S | N |
| EB861 | 2020 | Other-NS | E. cloacae | 4 | S | S | 0.25 | S | S | 0.125 | S | S | N |
| EB853 | 2020 | Other-NS | E. cloacae | 2 | S | S | 0.25 | S | HR | <0.06 | S | HR | Class A |
| EB862 | 2020 | Sterile | E. cloacae | 2 | S | S | 0.25 | S | HR | <0.06 | S | S | N |
| EB863 | 2020 | Respiratory | E. cloacae | >32 | R | HR | 16 | R | HR | 2 | S | HR | Class D |
| EB864 | 2020 | Other-NS | E. cloacae | 2 | S | S | 0.25 | S | HR | <0.06 | S | S | N |
| EB546 | 2021 | Respiratory | E. cloacae | >32 | R | R | >32 | R | S | >32 | R | HR | Class B |
| EB578 | 2021 | Urine | E. cloacae | 4 | S | S | 0.25 | S | S | 0.125 | S | S | N |
| EB555 | 2021 | Urine | E. cloacae | 1 | S | S | 0.125 | S | S | 0.125 | S | S | N |
| EB570 | 2021 | Urine | E. cloacae | 2 | S | S | 0.25 | S | S | 0.125 | S | HR | N |
| EB533 | 2021 | Urine | E. cloacae | 0.25 | S | S | 0.125 | S | S | <0.06 | S | S | N |
| EB550 | 2021 | Urine | E. cloacae | 0.125 | S | S | 0.125 | S | S | 0.125 | S | S | N |
| EB569 | 2021 | Sterile | E. cloacae | 1 | S | S | 0.25 | S | S | <0.06 | S | S | Class A |
| EB539 | 2021 | Sterile | E. cloacae | 0.5 | S | S | 0.125 | S | S | 0.25 | S | S | Class B |
| EB547 | 2021 | Other-NS | E. cloacae | 0.5 | S | S | 0.25 | S | S | <0.06 | S | S | N |
| EB563 | 2021 | Other-NS | E. cloacae | 0.25 | S | S | 0.5 | S | S | 0.125 | S | S | N |
| EB543 | 2021 | Urine | E. cloacae | 0.125 | S | HR | 0.25 | S | S | 0.125 | S | S | N |
| EB575 | 2021 | Urine | E. cloacae | 1 | S | S | 0.25 | S | S | 0.125 | S | S | N |
| EB566 | 2021 | Respiratory | E. cloacae | 1 | S | S | 0.25 | S | S | 0.125 | S | S | N |
| EB542 | 2021 | Respiratory | E. cloacae | 4 | S | S | 0.125 | S | S | <0.06 | S | S | N |
| EB576 | 2021 | Respiratory | E. cloacae | 0.5 | S | S | 0.25 | S | S | <0.06 | S | S | Class A |
| EB562 | 2021 | Urine | E. cloacae | 0.5 | S | S | 0.125 | S | S | 0.125 | S | S | N |
| EB574 | 2021 | Sterile | E. cloacae | 1 | S | S | 0.25 | S | S | <0.06 | S | S | N |
| EB573 | 2021 | Sterile | E. cloacae | 2 | S | S | 0.125 | S | S | 0.125 | S | S | N |
| EB545 | 2021 | Urine | E. cloacae | 4 | S | S | 0.25 | S | S | <0.06 | S | S | N |
| EB582 | 2021 | Urine | E. cloacae | <0.06 | S | S | <0.06 | S | S | <0.06 | S | S | N |
| EB577 | 2021 | Other-NS | E. cloacae | 1 | S | S | 0.25 | S | S | <0.06 | S | S | N |
| EB558 | 2021 | Sterile | E. cloacae | 4 | S | S | 0.25 | S | S | 0.5 | S | S | N |
| EB559 | 2021 | Urine | E. cloacae | <0.06 | S | S | 0.125 | S | S | <0.06 | S | S | N |
| EB538 | 2021 | Other-NS | E. cloacae | <0.06 | S | S | <0.06 | S | S | 0.125 | S | S | N |
| EB581 | 2021 | Other-NS | E. cloacae | 4 | S | S | 0.25 | S | S | 0.125 | S | S | N |
| EB551 | 2021 | Urine | E. cloacae | 1 | S | S | 0.25 | S | S | 0.125 | S | S | N |
| EB571 | 2021 | Sterile | E. cloacae | 4 | S | S | 0.25 | S | S | <0.06 | S | S | Class A |
| EB552 | 2021 | Sterile | E. cloacae | 4 | S | S | 0.25 | S | S | 0.25 | S | S | N |
| EB560 | 2021 | Sterile | E. cloacae | 0.25 | S | S | 0.125 | S | S | <0.06 | S | S | N |
| EB586 | 2021 | Urine | E. cloacae | 8 | S | S | 0.5 | S | S | 0.25 | S | S | N |
| EB568 | 2021 | Sterile | E. cloacae | 2 | S | S | 0.25 | S | S | 0.25 | S | S | N |
| EB583 | 2021 | Urine | E. cloacae | 0.25 | S | S | 0.125 | S | S | <0.06 | S | S | N |
| EB565 | 2021 | Urine | E. cloacae | 1 | S | S | 0.25 | S | S | 0.125 | S | S | N |
| EB548 | 2021 | Other-NS | E. cloacae | 1 | S | S | 0.25 | S | S | 0.5 | S | S | N |
| EB567 | 2021 | Urine | E. cloacae | 1 | S | S | 0.125 | S | S | 0.125 | S | S | N |
| EB580 | 2021 | Urine | E. cloacae | 4 | S | S | 0.25 | S | S | 0.125 | S | S | N |
| EB557 | 2021 | Respiratory | E. cloacae | 2 | S | S | 0.25 | S | S | 0.125 | S | S | N |
| EB561 | 2021 | Respiratory | E. cloacae | 1 | S | S | 0.25 | S | S | 0.125 | S | S | N |

Sterile culture source includes blood, bone, deep tissue/internal abscess, pleural fluid, peritoneal fluid, and other naturally sterile sites.

Other-NS culture source includes other non-sterile sources such as wounds, drainage, non-sterile tissue.

E. coli = Escherichia coli, KPN = Klebsiella pneumoniae, E. cloacae = Enterobacter cloacae complex

BMD MIC = broth microdilution minimum inhibitory concentration

AST Int = antimicrobial susceptibility testing interpretation

PAP = population analysis profile

S= susceptible, I=Intermediate, R=Resistant; HR = Heteroresistant

Class A: Ambler class of carbapenemases that includes members of the SME, IMI, NMC, GES, and KPC families.

Class B: Ambler class of carbapenemases that encompass metallo-β-lactamases belong to the NDM, IMP, VIM, SPM, GIM, and SIM families.

Class D: Ambler class of carbapenemases that encompass oxacillinases (or OXA-type β-lactamases

Table S2: Proportion of carbapenem-resistant Enterobacterales isolates susceptible or heteroresistant/resistant, as determined by population analysis profile, to newer β-lactamase/β-lactamase inhibitors by culture site.

|  | CAZ/AVI | | IPM/REL | | MEM/VAB | |
| --- | --- | --- | --- | --- | --- | --- |
| Culture Site | S  n (%) | HR/R  n (%) | S  n (%) | HR/R  n (%) | S  n (%) | HR/R  n (%) |
| Urine (n=154) | 138 (90) | 16 (10) | 130 (84) | 24 (16) | 143 (93) | 11 (7) |
| Sterile* (n=87) | 72 (83) | 15 (17) | 76 (87) | 11 (12) | 85 (98) | 2 (2) |
| Respiratory (n=44) | 38 (86) | 6 (14) | 35 (80) | 9 (20) | 39 (89) | 5 (11) |
| Other** (n=42) | 37 (88) | 5 (12) | 35 (83) | 7 (16) | 41 (98) | 1 (2) |

*Sterile = Blood, bone, deep tissue/internal abscess, pleural fluid, peritoneal fluid, and other naturally sterile sites

**Other = Other non-sterile culture sites such as wounds, drainage, non-sterile tissue

CAZ/AVI = ceftazidime-avibactam; IPM/REL = imipenem-relebactam; MEM/VAB = meropenem-vaborbactam

S = Susceptibility; HR/R = Heteroresistant and/or Resistant

Table S3: Proportion of carbapenem-resistant Enterobacterales isolates susceptible (S), intermediate (I), heteroresistant (HR), or resistant to newer β-lactamase/β-lactamase inhibitors by broth microdilution compared to population analysis profile in two academic hospitals in Atlanta, Georgia, 2016-2021

(a) Ceftazidime- avibactam (CAZ/AVI)

|  | Broth Microdilution | | Population Analysis Profile | |
| --- | --- | --- | --- | --- |
| CRE isolate species | S  n (%) | R  n (%) | S  n (%) | HR/R  n (%) |
| *E. coli* (n=53) | 50 (96) | 3 (6) | 49 (93) | 4 (8) |
| *K. pneumoniae* (n=123) | 116 (94) | 7 (6) | 101 (82) | 22 (18) |
| *E. cloacae* (n=151) | 148 (98) | 3 (2) | 135 (89) | 16 (11) |
| Total (n=327) | 314 (96) | 13 (4) | 285 (87) | 42 (13) |

(b) Imipenem-relebactam (IPM/REL)

|  | Broth Microdilution | | | Population Analysis Profile | |
| --- | --- | --- | --- | --- | --- |
| CRE isolate species | S  n (%) | I  n (%) | R  n (%) | S  n (%) | HR/R  n (%) |
| *E. coli* (n=53) | 46 (87) | 3 (5.5) | 4 (7.5) | 48 (91) | 5(9) |
| *K. pneumoniae* (n=123) | 109 (88.6) | 6 (4.9) | 8 (6.5) | 87 (70.7) | 36 (29.3) |
| *E. cloacae* (n=151) | 142 (94) | 7 (4.6) | 2 (1.4) | 141 (93.4) | 10 (6.6) |
| Total (n=327) | 297 (90.8) | 16 (4.9) | 14 (4.3) | 276 (84.4) | 51 (16) |

(c) Meropenem-vaborbactam (MEM/VAB)

|  | Broth Microdilution | | | Population Analysis Profile | |
| --- | --- | --- | --- | --- | --- |
| CRE isolate Species | S  n (%) | I  n (%) | R  n (%) | S  n (%) | HR/R  n (%) |
| *E. coli* (n=53) | 49 (92.5) | 1 (2) | 3 (5.5) | 49 (92.5) | 4 (7.5) |
| *K. pneumoniae* (n=123) | 112 (91.1) | 3 (2.4) | 8 (6.5) | 112 (91.1) | 11 (8.9) |
| *E. cloacae* (n=151) | 150 (99.4) | 0 (0) | 1 (0.6) | 147 (97.3) | 4 (2.7) |
| Total (n=327) | 311 (95.1) | 4 (1.2) | 12 (3.7) | 308 (94.2) | 19 (5.8) |
